# Charge Storage Mechanisms in Redox-Active Polymer Brushes

**DOI:** 10.1021/acs.macromol.5c03354

**Published:** 2026-02-02

**Authors:** Oleg Rud, Sergii Chertopalov, Oleg Borisov

**Affiliations:** † Department of Physical and Macromolecular Chemistry, Faculty of Science, Charles University, Prague 128 00, Czech Republic; ‡ 86889 Institute of Physics of the Czech Academy of Sciences, Prague 182 00, Czech Republic; § CNRS-UPPA, Institut des Sciences Analytiques et de Physico-Chimie pour L’Environnement et Les Matériaux, Pau 64053, France

## Abstract

Electroconductive polymer brushes grafted to conductive electrodes are investigated as model electrodes for aqueous supercapacitors using the Scheutjens–Fleer self-consistent field (SF-SCF) framework. The model self-consistently resolves polymer conformations, ion partitioning, and redox-mediated electron hopping under applied potentials (0–0.7 V). We show that solvent quality and grafting density govern brush swelling and counterion uptake, thus shaping the charge-potential response. In a good solvent, brushes provide volumetric charge storage throughout a swollen layer, while in a poor solvent, charging drives a collapsed-to-swollen transition that produces sharp capacitance peaks. During this transition, the differential capacitance reaches 15–30 F/m^2^, an order of magnitude higher than the bare-electrode baseline. These results demonstrate how redox-active electroconductive brushes integrate electric double-layer and pseudocapacitive mechanisms, providing design principles for polymer-brush-modified electrodes in both supercapacitors and ion-selective membranes.

## Graphical abstract

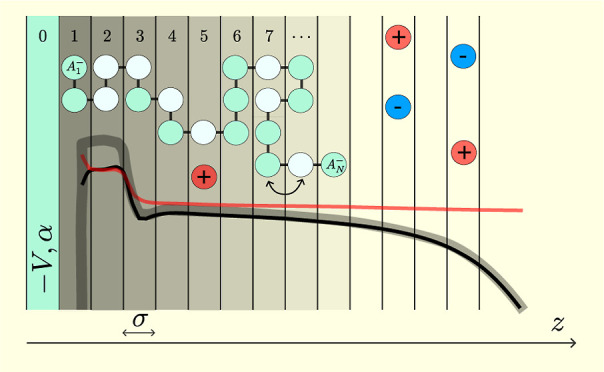

## Introduction

### Supercapacitors

Supercapacitors (SCs), or electrochemical capacitors, store charge electrostatically in the electric double layer (EDL) at electrode–electrolyte interfaces, a picture rooted in the Helmholtz model and refined by Gouy, Chapman, and Stern.^1,2^ In contrast to batteries, SCs deliver rapid charge–discharge, high power density, and long cycle life, which is advantageous for hybrid electric vehicles, grid buffering of renewables, and portable electronics.^3^ Commercial EDL devices typically employ porous carbon electrodes with specific surface areas of 1000–2000 m^2^/g and surface charge densities on the order of 0.1–0.3 C/m^2^.^4^ The primary limitation remains energy density, motivating materials that add redox-active charge storage without sacrificing charge–discharge rate capability.

### Pseudocapacitance and Hybrid Mechanisms

Beyond pure EDL storage, fast, reversible faradaic reactions add pseudocapacitance that can substantially raise total capacitance, provided that ion transport and electron transfer are not rate-limiting.^2,5^ Hybrid supercapacitors combine EDL and faradaic components to pair high power with improved energy density.^6,7^ Material optimization spans high-area carbons (activated carbon, graphene, nanotubes, etc.) for ion access and conductivity,^8,9^ redox-active metal oxides,^10^ and carbon–polymer nanocomposites that blend both mechanisms.^11^

### Performance of Electroconductive Polymers

Electroconductive polymers such as polyaniline (PANI), polypyrrole (PPy), and poly­(3,4-ethylenedioxythiophene) (PEDOT) are well-established pseudocapacitive materials. For instance, in acidic aqueous electrolytes, PPy typically delivers approximately 510 F/g.^12^ Composites push performance further: PANI with activated carbon yields approximately 300 F/g,^13^ PANI–fullerene reaches up to 813 F/g,^14^ and hybrids including PANI/graphene,^15^ PANI/MnO_2_,^16^ and other metal–oxide combinations^10,17^ report maxima near 1360 F/g.^11^ Assuming 1000–2000 m^2^/g porous electrodes, these mass-specific values correspond to roughly 0.15–1.3 F/m^2^. A practical hurdle, however, is that many electroconductive polymers are intrinsically hydrophobic, which restricts ion access and limits performance in water-based electrolytes.^18,19^ Hydrophilization—for example, water-compatible PANI–cellulose complexes or alkyl-substituted PPy—improves processability and opens ionic pathways.^20,21^

### Materials and Design Strategies

Extending this idea, hydrogels or brushes of electroconductive polymers tethered to conductive supports create soft and permeable architectures suitable for high-speed, durable operation.^22–24^ Here, we focus on electroconductive polymer brushes—polymer chains covalently tethered to a conductive substrate—as a means to couple EDL and pseudocapacitance within a single, ion-accessible nanostructure. By tuning grafting density, chain length, and segment chemistry, brushes can regulate ion partitioning, reduce tortuosity, and promote electron percolation along the backbone.^22,24^ This motivates the computational modeling developed in the following to quantify how brush architecture and hydrophilicity map to capacitance under applied potential.

### Insights from Battery Technology

Strategies developed for battery electrodes offer valuable insight into improving ion regulation in supercapacitors. For example, sodium-ion batteries exploit abundant Na^+^ ions but face challenges due to their larger ionic radius and dendrite formation. Polymer electrolytes have been shown to alleviate these problems by improving ion mobility and stabilizing the metal–polymer interface.^25^ Similarly, in aqueous zinc–ion batteries, hydrogel-based electrodes and MXene–cellulose nanofibril composites suppress dendrite growth and improve cycling stability.^26^ Inspired by these approaches, we extend these ion-regulation concepts to hydrophilic electroconductive polymer brushes, where controlled ion partitioning and electron transport are expected to enhance capacitance in Na^+^-based supercapacitors.

### Ion Partitioning in Polyelectrolytes

Previous studies of polyelectrolyte systems—both brushes and hydrogels—have shown that their charged networks can effectively regulate ion accessibility and charge distribution, offering useful design principles for electrochemical interfaces.^27–29^ Brush architecture strongly affects ion partitioning, swelling, and screening behavior in electrolyte environments. Despite these insights, the coupling between electronic conductivity and ion doping in hydrophilic electroconductive polymer brushes, especially in aqueous electrolytes such as NaCl, remains poorly understood. A comprehensive theoretical framework that captures these interdependent effects is needed to predict their electrochemical performance.

### Charge Transport in Conductive Polymers

Efficient charge transport within electroconductive polymers is essential for maintaining high-rate performance in supercapacitors. At the molecular level, conductivity arises primarily through polaron hopping along and between polymer chains, as established in the seminal work of Heeger and co-workers.^30^ Ab initio simulations by Zahabi et al. revealed that structural distortions in PEDOT promote polaron delocalization and facilitate electronic conduction between monomer units.^31^ On larger length scales, Monte Carlo simulations by Ihnatsenka et al. showed that electronic transport in disordered electroconductive polymers (e.g., doped PANI) strongly depends on the degree of structural disorder and carrier concentration.^32^ Together, these studies highlight how molecular geometry, electronic coupling, and disorder govern the charge mobility that must be incorporated into theoretical models of hydrophilic electroconductive polymer brushes.

### Research Hypothesis

We hypothesize that electroconductive polymer brushes tethered to metallic electrodes can substantially enhance supercapacitor performance by combining electric double-layer and faradaic charge-storage mechanisms within a single, ion-accessible nanostructure. Upon charging, the grafted layer is expected to swell and accumulate counterions, facilitating both ion doping and charge delocalization along the polymer backbone. This synergy between ionic and electronic transport should yield a higher specific capacitance and improved cycling stability compared to planar or nonconductive interfaces.

### SF-SCF Modeling Framework

To test this hypothesis, we employ the Scheutjens–Fleer self-consistent field (SF-SCF) approach to model hydrophilic electroconductive polymer brushes in aqueous NaCl electrolytes under applied potentials ranging from 0 to 0.7 V. The SF-SCF framework captures polymer conformations, ion partitioning, electrostatic interactions, and redox-induced charge regulation within a self-consistent mean-field description.^33^ By systematically varying grafting density, chain length, dielectric properties, and electron-hopping strength, we analyze how brush architecture governs ion accessibility and charge storage. The results establish a theoretical foundation for the rational design of polymer-brush-modified electrodes, paving the way toward next-generation, high-efficiency supercapacitors. To our knowledge, this work provides the first SF-SCF treatment of redox-active electroconductive polymer brushes under applied potential, quantifying how redox charge regulation, ion partitioning, and brush swelling contribute to capacitance. This approach extends prior SF-SCF studies of weak polyelectrolyte brushes and hydrogels^27,34,35^ by explicitly coupling the redox state of segments with the electrode potential through intrachain electron hopping, introducing a spatially dependent charge regulation mechanism.

## Model Description

### Theoretical Foundation

Electroconductive polymer brushes tethered to a planar metallic electrode in an aqueous NaCl electrolyte are modeled using a one-dimensional SF-SCF approach. The model resolves spatial distributions of polymer segments, mobile ions, and electrostatic potential perpendicular to the electrode surface (Figure 1), capturing the interplay between brush architecture, electron hopping, and ion partitioning. The surface charge density of the metallic electrode is prescribed as an input parameter, while the resulting electrostatic potential profile, ψ­(*z*), emerges self-consistently from the SF-SCF calculations.

**Figure 1:**
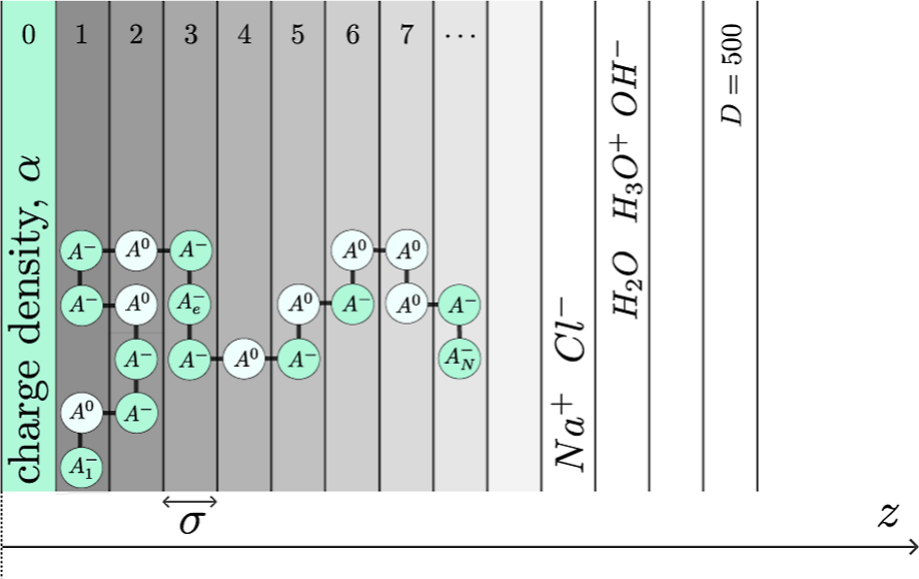
Schematic representation of the one-dimensional SF-SCF model geometry for a hydrophilic electroconductive polymer brush grafted to a planar electrode in aqueous electrolyte.

### Solvent Quality and Flory–Huggins Parameter

The Flory–Huggins parameter χ quantifies polymer–solvent compatibility and is a key control parameter for swelling. χ = 0 corresponds to an athermal solvent (good solvent), while χ = 0.5 denotes the theta-point. For intrinsically hydrophobic electroconductive polymers, χ is expected to be much larger. Using the solubility parameter approach,^36^ we estimated aqueous χ values of approximately 5.18 for polyaniline (PANI), 5.56 for polypyrrole (PPy), and 3.49 for PEDOT:PSS (based on their respective solubility parameters δ). These high values confirm the poor solvent conditions (χ = 4) typical for aqueous dispersions. However, modifying the solvent (e.g., PANI in ethanol yields χ approximately 0.82) or doping can improve compatibility. To capture this broad range, we employ representative effective parameters χ = 0, 2, and 4, spanning from good solvent (χ = 0, mimicking compatible states) to moderately (χ = 2) and strongly poor (χ = 4) solvent conditions.

### Lattice Geometry and Discretization

The simulation domain is divided into *D* = 500 planar layers indexed by *z* = 1, 2, ..., *D* (Figure 1). The metallic electrode is located at *z* = 0, with the polymer brush grafted at *z* = 1. Layers *z* > 1 contain the brush and aqueous NaCl electrolyte. Each lattice layer has a thickness of σ = 0.35 nm. This value is close to the molecular size of water (approximately 0.31 nm) and approximately half of the Bjerrum length in water, a common choice in coarse-grained discretizations that balances spatial resolution and numerical stability.

Because each lattice site represents a volume σ^3^, a fully occupied layer corresponds to the reference concentration

1 $$\varphi^{ref}=\frac{1}{\sigma^{3}N_{A}}\approx 38.7mol/L$$

All ionic and molecular concentrations in the SF-SCF formulation are therefore expressed as volume fractions φ_*i*_/φ^ref^, consistent with the lattice-based definition of chemical potentials.

### Electrode and Brush Architecture

The metallic electrode is modeled as a fixed monolayer of charged lattice sites, each carrying an effective charge α that specifies the surface charge density. The electrode thus acts as a boundary condition for the charge density at *z* = 0.

Each polymer chain consists of *N* = 200 monomeric segments: a grafting unit (*A*_1_), the (*N* – 2) backbone monomers (*A*), and a terminal unit (*A*_*N*_). Chains are tethered at layer *z* = 1 via the *A*_1_ segment. The grafting density ϕ is controlled by the density of *A*_1_ segments in the surface layer and can be varied between 0 and 0.4 chains/nm^2^. In this work we focus on ϕ = 0.20 chains/nm^2^, where the lateral spacing between grafting points is sufficiently small for the brush to operate in the strong-brush (quasi-neutral) regime. Chain stretching is then dominated by excluded-volume interactions imposed by the high grafting density, and electrostatic charging acts as an additional perturbation on an already strongly stretched brush. This regime is typical for dense synthetic brushes and enables a clean separation between conformational stretching due to crowding and charge regulation due to redox processes.

### Redox States and Charge Mobility

Each brush monomer can exist in a neutral (*A* ^0^) or reduced (*A* ^–^) redox state. Electron exchange between the grafting segment and the metallic electrode is represented by

2 $$A_{1}^{0}+e^{-}\;⇌\;A_{1}^{-}$$

and is characterized by an equilibrium constant *K*_1_ = 1. This choice corresponds to fast, reversible electron transfer at the grafting site, ensuring that its redox state is set directly by the applied electrode potential.

Charge redistribution along the polymer backbone occurs via intrachain electron hopping, modeled by the redox exchange

3 $$A^{-}+A_{1}^{0}\;⇌\;A^{0}+A_{1}^{-}$$

which transfers electronic charge between backbone segments and the grafting unit. For simplicity, the same equilibrium constant (*K* = *K*_1_ = 1) is used for this process, reflecting instantaneous equilibrium redox redistribution (rather than kinetics) along the chain. This assumption focuses the model on equilibrium charge distributions rather than kinetic limitations in electron transfer.

### Electrolyte and pH Balance

The aqueous electrolyte is modeled as a mixture of sodium and chloride ions at bulk concentration *c*_s_ = φ_Na^+^_, with the chemical potential of Na^+^ set by

4 $$\mu_{{Na}^{+}}=k_{B}T\;\ln\left(\varphi_{{Na}^{+}}/\varphi^{ref}\right)$$

in addition to the added salt, the solution contains the intrinsic ions of water autoprotolysis, H_3_O^+^ and OH^–^, which satisfy the equilibrium condition

5 $$2H_{2}O\;⇌\;H_{3}O^{+}+{OH}^{-},,K_{w}=10^{-14}\;{\left(mol/L\right)}^{2}$$

These autoionization species enable the system to adjust its local pH in response to electrostatic and redox processes and, together with the salt ions, ensure global electroneutrality throughout the SF-SCF calculation.

### Scheutjens–Fleer Self-Consistent Field Method

The SF-SCF approach^37–41^ is a lattice-based mean-field theory in which explicit particle interactions are replaced by spatially varying effective fields. For planar geometries, the system reduces to a one-dimensional lattice perpendicular to the electrode, with all species represented by layer-resolved density profiles φ_*i*_(*z*).

Mobile ions respond to the local electrostatic potential ψ­(*z*) according to Boltzmann statistics

6 $$\varphi_{i}\left(z\right)=\varphi_{i}^{bulk}\;\exp\left[-\frac{q_{i}e\psi \left(z\right)}{k_{B}T}\right]$$

and global electroneutrality requires

7 $$\sum_{i}q_{i}\int \varphi_{i}\left(z\right)\;dz=0$$

where the index *i* runs over all charged components of the system, including mobile ions, charged polymer segments, and the fixed surface charges of the electrode. The electrostatic potential satisfies Poisson’s equation

8 $$\frac{d^{2}\psi \left(z\right)}{dz^{2}}=-\frac{e}{\varepsilon \varepsilon_{0}}\;\sum_{i}q_{i}\varphi_{i}\left(z\right)$$

with ε_0_ the vacuum permittivity and ε = 80 the dielectric constant of water.

The total mean-field potential acting on species *i* is

9 $$u_{i}\left(z\right)=u^{\prime }\left(z\right)+u_{i}^{short}\left(z\right)+u_{i}^{el}\left(z\right)$$

where *u*′(*z*) enforces incompressibility, *u*_*i*_^short^(*z*) accounts for short-range interactions through the Flory–Huggins parameters χ_*ij*_, and *u*_*i*_^el^(*z*) is the electrostatic contribution from ψ­(*z*).

Polymer connectivity is implemented by lattice random walks: each monomer occupies a single lattice site, excluded volume is enforced, and the segment distributions are obtained from forward–backward propagators satisfying the discrete Edwards diffusion equation.^42,43^ Grafting is realized by fixing the first segment (*A*_1_) at the surface layer. In this lattice formulation, all densities φ_*i*_(*z*) represent normalized probabilities of finding species *i* in layer *z*, subject to the local incompressibility condition. Self-consistency is achieved by iteratively updating densities and potentials until convergence.

Extensions of SF-SCF incorporate ionization equilibria in weak polyelectrolytes^34,35^ and have been extensively applied to brushes, stars, and hydrogels.^27,44–46^ Here we use the framework to describe redox-active brushes in aqueous electrolytes, including electron hopping via reaction (3).

### Computational Implementation

All calculations were performed using the SFBox package. The system is initialized with bulk ion concentrations and Gaussian chain statistics. Boundary conditions are imposed as a fixed surface charge density α at the electrode (*z* = 0) and ψ­(*D*) = 0 at the bulk boundary.

Each iteration consists of: (i) updating mean-field potentials *u*_*i*_(*z*) from the current densities; (ii) computing polymer segment distributions using forward–backward propagation; (iii) solving Poisson’s equation for ψ­(*z*); and (iv) renormalizing all densities to satisfy incompressibility. Convergence is achieved when all density changes fall below 10^–7^.

Simulations were conducted over a broad range of grafting densities (0.01–0.4 chains/nm^2^), applied potentials (0–0.7 V), and Flory–Huggins parameters (χ = 0, 2, 4). Unless stated otherwise, the figures and discussion correspond to the representative dense-brush case ϕ = 0.20 chains/nm^2^, where excluded-volume interactions dominate chain stretching. From the converged profiles φ_*i*_(*z*) and ψ­(*z*), we compute the accumulated ionic charge *Q*, the surface potential *V*, and the differential capacitance *C* = d*Q*/d*V*. All reported capacitance values are normalized to the geometric surface area of the planar substrate.

## Results and Discussion

### Electric Double Layer at a Bare Electrode

We first examine ion distributions near a planar electrode in the absence of a polymer brush. For a bare planar electrode, the simulations reproduce the classical EDL structure described by Poisson–Boltzmann theory. Figure 2a shows the density profiles of Na^+^ and Cl^–^ ions for three salt concentrations *c*_s_.

**Figure 2:**
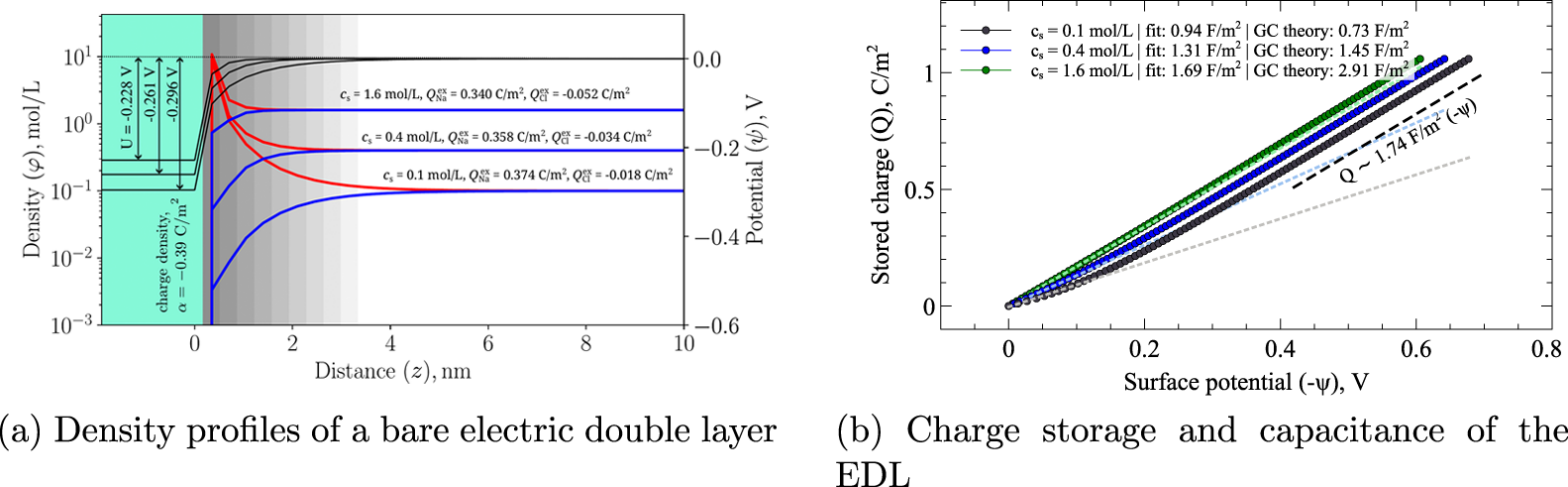
(a) Number-density profiles of Na^+^ (red) and Cl^–^ (blue) near a negatively charged planar electrode with σ = – 0.39 C/m^2^ for *c*_s_ = 0.1, 0.4, and 1.6 mol/L. Black curves show electrostatic potentials with annotated *Q*_*i*_^ex^ and *U*. (b) Stored ionic charge *Q* vs surface potential. Symbols show simulation data with linear fits at low potential. The dashed line indicates the common high-potential slope approached by all three *Q*(ψ) curves, corresponding to a Helmholtz capacitance of *C* approximately 1.7 F/m^2^ (eq 14 with ε approximately 80 and δ approximately 0.4 nm).

For *c*_s_ = 0.4 mol/L and a negatively charged electrode (α = – 0.39 C/m^2^), counterions (Na^+^) accumulate near the surface, yielding an excess adsorption

10 $$Q_{{Na}^{+}}^{ex}=e\int_{0}^{\infty }\;\left[\varphi_{{Na}^{+}}\left(z\right)-\varphi_{{Na}^{+}}^{bulk}\right]\;dz\approx 0.358C/m^{2}$$

Co-ions (Cl^–^) are slightly depleted

11 $$Q_{{Cl}^{-}}^{ex}\approx -0.034C/m^{2}$$

and the sum of counterion and co-ion excesses compensates the imposed surface charge, ensuring overall electroneutrality.

For reference, the corresponding Gouy–Chapman length

12 $$\lambda_{GC}=\frac{\varepsilon \varepsilon_{0}k_{B}T}{e\left|\sigma \right|}\approx 0.46nm$$

quantifies the characteristic thickness of the diffuse layer associated with α = – 0.39 C/m^2^. The Gouy–Chapman length associated with this surface charge is smaller than the lattice spacing; this small λ_GC_ reflects the strong near-surface electric field and the correspondingly high counterion density in the first few layers. In the presence of added salt, however, the overall decay of the ion profiles is governed by the Debye length (1–3 nm for the salinities considered), so the counterion density decreases over several nanometers rather than over λ_GC_ alone.

### Key Features of the Electric Double Layer

Figure 2 summarizes the main characteristics of the electric double layer (EDL):

- Diffuse layer scaling. For a symmetric 1:1 electrolyte, the Debye length is $\lambda_{D}\approx 0.304/\sqrt{c_{s}}$ nm, decreasing from about 0.96 nm at *c*_s_ = 0.10 mol/L to 0.24 nm at *c*_s_ = 1.60 mol/L. Accordingly, the surface potential decreases slightly (from −0.296 V to −0.228 V) with increasing salinity due to stronger electrostatic screening. In all cases, ion densities differ from bulk only within a few Debye lengths (roughly 1–3 nm).
- Electroneutrality. The excess adsorptions of Na^+^ and Cl^–^ ions compensate the imposed surface charge density, $Q_{{Na}^{+}}^{ex}-Q_{{Cl}^{-}}^{ex}=\alpha =-0.39$ C/m^2^, ensuring overall electroneutrality.
- Monotonic ion profiles. Counterion densities decay monotonically away from the electrode, while co-ion densities rise monotonically toward the bulk. This behavior is characteristic of Poisson–Boltzmann theory, which neglects ion–ion correlations and steric effects; real EDLs often show oscillations, over-screening, or layering that are absent in mean-field models.
- Potential drop. The electrostatic potential decays smoothly without an explicit Helmholtz layer. As a result, most of the potential drop is confined to the first few lattice layers, and the calculated capacitance is dominated by this Helmholtz separation and only weakly sensitive to the bulk salt concentration, *c*_s_.

### Capacitance of the Electric Double Layer

The differential capacitance is defined as

13 $$C_{d}=\frac{dQ}{d\psi }$$

where *Q* is the total excess ionic charge stored in the double layer and ψ is the surface potential. In the classical Helmholtz picture, the double layer behaves as a parallel-plate capacitor of thickness δ

14 $$C_{d}=\frac{\varepsilon_{0}\varepsilon_{r}}{\delta }$$

which for bulk-water permittivity ε_r_ approximately 80 and a molecular separation δ between 0.35 and 0.5 nm yields *C*_d_ between 1.7 and 2.0 F/m^2^. Experimental capacitances of planar metal/aqueous interfaces are typically lower, 0.2–0.4 F/m^2^,^2^ due to dielectric saturation, oxide films, and specific adsorption that reduce the effective permittivity of the compact layer. Our simulated high-potential slope (nearly 1.74 F/m^2^) therefore approaches this ideal Helmholtz limit rather than the reduced values observed experimentally.

Figure 2b shows the simulated *Q*(ψ) dependencies for three salt concentrations. At low surface potentials, the *Q*(ψ) curves exhibit salinity-dependent initial slopes that follow the Gouy–Chapman (GC) prediction for a symmetric 1:1 electrolyte

15 $$C_{GC}=\frac{\varepsilon \varepsilon_{0}}{\lambda_{D}}\cosh\left(\frac{e\psi_{0}}{2k_{B}T}\right),,\lambda_{D}=\sqrt{\frac{\varepsilon \varepsilon_{0}k_{B}T}{2c_{s}e^{2}}}$$

in the Debye–Hückel limit (eψ_0_ ≪ *k*_B_ *T*), the capacitance simplifies to

16 $$C_{GC}\approx \frac{\varepsilon \varepsilon_{0}}{\lambda_{D}}\propto \sqrt{c_{s}}$$

which explains the increase in slope with salinity observed in the low-voltage region.

At higher potentials, the three *Q*(ψ) curves become parallel and approach a common asymptotic slope (indicated by the dashed line in Figure 2b). This reflects counterion saturation near the electrode: once ions reach their maximum packing density, further increases in potential no longer raise the local concentration. The classical Poisson–Boltzmann model cannot capture steric exclusion or dielectric saturation and therefore overestimates charge at high ψ. In contrast, the present mean-field treatment naturally reproduces this limiting-capacitance regime, in line with modified Poisson–Boltzmann (finite-size) theories.

### Structure of Polymer-Brush-Modified Electrodes

We now extend this analysis to electrodes coated with electroconductive polymer brushes. Polymer brushes used in experiments typically contain *N* = 50–1000 monomer units per chain, depending on the synthesis route and polymer chemistry.^48^ For electroconductive polymers such as PANI, the repeat-unit length is *b* approximately 0.50 nm.^49^ The grafting density ϕ controls the lateral spacing between chains and therefore determines the degree of chain stretching.

### Transition to Brush-Modified Interfaces

Because polyelectrolyte and redox-active brushes do not follow the simple mushroom-to-brush crossover of neutral polymers,^48,50^ we do not assign numerical thresholds for this transition. Instead, we treat ϕ as a tunable geometric parameter that establishes the overall extension of the brush.

Compared with planar PANI films, grafted brushes provide a much larger ion-accessible volume because stretched chains produce a permeable, swollen layer. This openness improves solvent uptake and counterion accessibility, in accordance with experimental observations for hydrophilized PANI systems.^15^

### Ion and Segment Distributions

Figure 3 shows the SF-SCF segment and ion distributions for a hydrophilic electroconductive polymer brush with chain length *N* = 200 and grafting density ϕ = 0.20 chains/nm^2^ in 0.4 mol/L NaCl, evaluated at two electrode charge densities α. Several features stand out:

- Brush profile. The total monomer density (gray line) extends to approximately 40 nm and is nearly uniform throughout the interior, with a sharp decay near the edge of the brush, typical for strongly stretched chains.^51^
- End-segment distribution. Chain ends (green dashed line) accumulate near the outer interface. As the surface charge density α increases, this density peak shifts outward, reflecting additional electrostatic stretching of chains driven by charging of the brush backbone.
- Charge regulation. Each monomer can switch between a neutral and a reduced (negatively charged) redox state. At α = 0.13 C/m^2^, only a small fraction of segments becomes negatively charged, whereas at α = 0.39 C/m^2^ the density of reduced segments increases substantially. This demonstrates self-regulated redox charging of the backbone driven by the electrode potential.
- Ion penetration. Sodium ions (red line) penetrate deeply inside the brush and closely follow the charged-segment distribution. Excess Na^+^ increases from $Q_{{Na}^{+}}^{ex}=1.34$ to 2.943 C/m^2^ as α increases from 0.13 to 0.39 C/m^2^. By contrast, Cl^–^ ions are largely excluded due to Donnan partitioning caused by negatively charged brush segments, with only a weak concentration inside the brush and a modest excess depletion.
- Potential profile. The electrostatic potential (black dashed line) decays gradually across the brush. A higher α deepens the potential drop (−0.118 vs – 0.267 V), promoting stronger counterion uptake throughout the brush layer.

**Figure 3:**
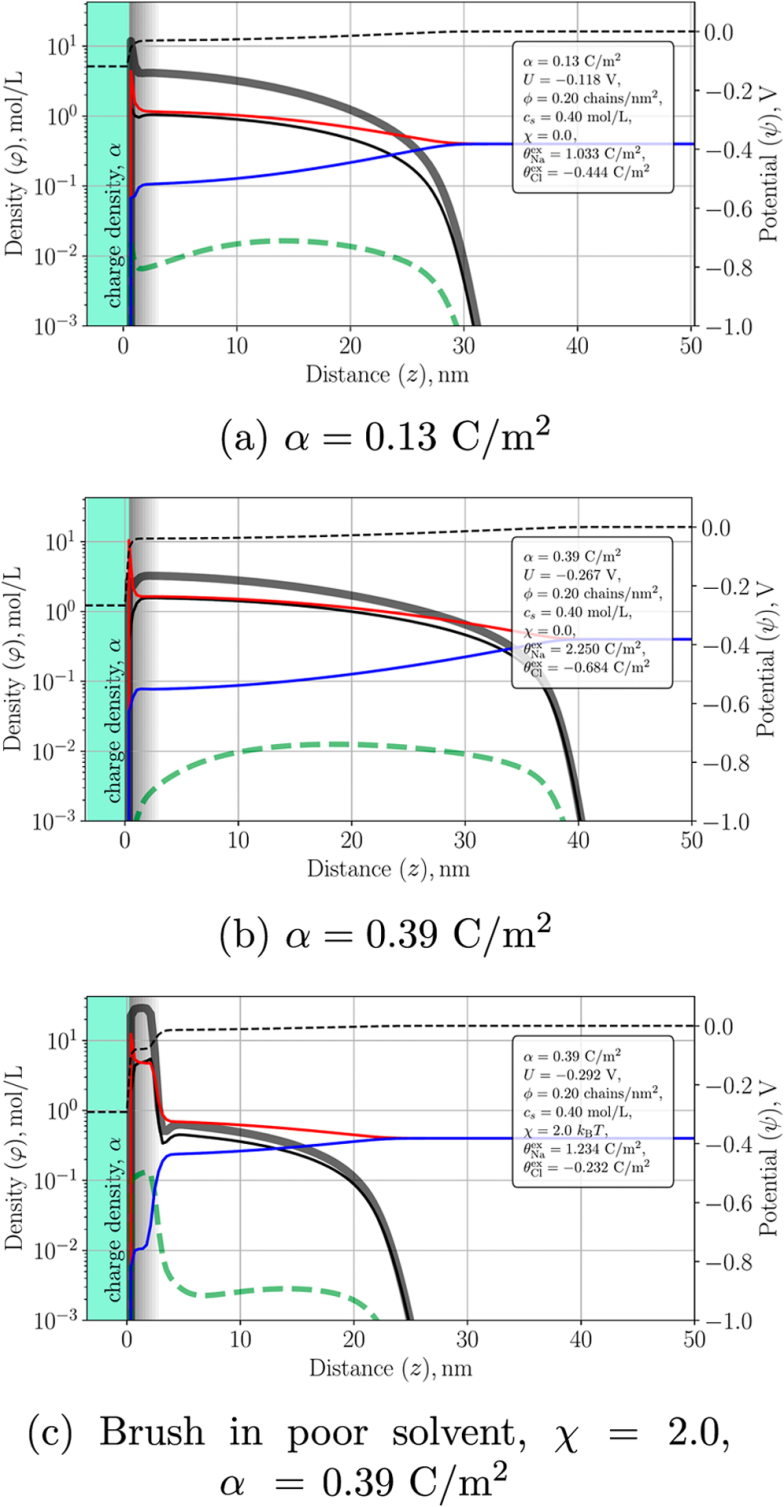
Simulated number-density profiles of Na^+^ (red), Cl^–^ (blue), total polymer segments (gray), charged segments (black), and chain ends (green dashed) for a polymer brush with chain length *N* = 200 and grafting density ϕ = 0.20 chains/nm^2^ in 0.4 mol/L NaCl. Panels (a) and (b) correspond to a good solvent (χ = 0) at two surface charge densities α, while panel (c) shows the same brush in a moderately poor solvent (χ = 2) at α = 0.39 C/m^2^.

### Charge-Storage Amplification

The brush acts as a volumetric ion reservoir: instead of charge being confined to a narrow Helmholtz layer, counterions permeate the entire brush thickness. At α = 0.39 C/m^2^, the net ionic excess is

17 $$Q=Q_{{Na}^{+}\;}^{ex}-Q_{{Cl}^{-}}^{ex}=2.943-\left(-0.895\right)=3.838C/m^{2}$$

nearly an order of magnitude larger than the bare-electrode charge density. This enhancement arises because the total interfacial charge comprises not only the surface charge α but also the charge carried by the redox-active brush segments. In the present model, the brush can sustain a much larger internal charge than the bare surface, and electroneutrality is maintained by volumetric counterion uptake throughout the brush layer. Increasing either the grafting density or the chain length would further amplify this effect, demonstrating how volumetric ion uptake and redox charging combine to augment the classical double-layer mechanism.

### Poor Solvent Conditions

As discussed already most of electroconductive polymers are intrinsically hydrophobic, thus solvent quality plays a central role in determining brush structure and therefore its capacitance. Figure 3c shows the same brush in a moderately poor solvent (χ = 2). Here the brush develops a dense inner layer near the substrate, with segment densities reaching approximately 55 mol/L and minimal solvent penetration. Beyond this collapsed domain lies a more dilute outer region extending into the electrolyte. The end-segment distribution becomes bimodal, reflecting coexistence of chains trapped in the collapsed layer and chains extending into solution. Such microphase-separated conformations arise from the competition between hydrophobic attraction and electrostatic swelling.^27^ As α increases, the collapsed region gradually erodes and the brush transitions toward a uniformly swollen state. This qualitative change in the conformational state of the brush—specifically the redox-induced collapse-swelling transition—is a unique feature of the brush architecture that cannot be captured by classical electric double-layer models.

### Origins of Enhanced Storage

The substantial counterion uptake and redox-induced swelling observed in polymer-brush-modified electrodes indicate strong potential for enhanced charge storage. As illustrated in Figure 3, the concentration of mobile ions inside the brush is comparable to or larger than the density of charged monomer units, placing the system in a salt-dominated regime. In poor solvent, this imbalance helps explain the stratified brush structure: a dense, solvent-poor inner region persists until the brush charge becomes large enough for electrostatic swelling to overcome hydrophobic attraction.

The next subsection quantifies this enhancement through differential capacitance and examines its dependence on salinity and solvent quality.

### Charge-Potential Response and Capacitance

#### Capacitance of a Polymer-Brush-Modified Electrode

Figure 4 shows the total excess ionic charge *Q* stored within the polymer brush as a function of the surface potential ψ for two salt concentrations (*c*_s_ = 0.4 and 1.6 mol/L) and several solvent qualities (χ = 0.0, 2.0, and 4.0) at a fixed grafting density ϕ = 0.20 chains/nm^2^. The thin straight lines represent the charge-potential response of a bare electrode in the same electrolyte (identical to the *c*_s_ = 0.4 and 1.6 mol/L curves in Figure 2b). Their common slope corresponds to the Helmholtz capacitance given by eq 14 and provides the empty-layer baseline against which the amplified charging of the brush can be compared.

**Figure 4:**
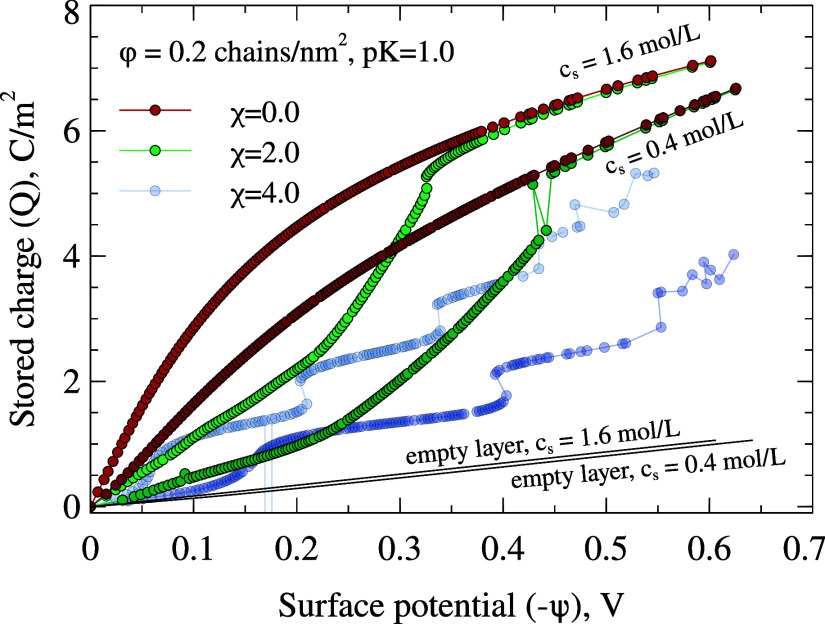
Total excess ionic charge *Q* within the brush layer as a function of surface potential ψ for different solvent qualities (χ = 0.0 and 2.0) and two bulk salinities (*c*_s_ = 0.4 and 1.6 mol/L). The thin straight “empty-layer” lines are the bare-electrode *Q*(ψ) curves from Figure 2b.

#### Role of Salinity and Hydrophobicity

In good solvent (χ = 0), the brush is swollen and supports volumetric counterion uptake. Accordingly, the initial slope of *Q*(ψ) is steeper than that of the bare electrode, indicating enhanced differential capacitance. At high ψ, the brush becomes saturated with counterions and the slope approaches the baseline.

In moderately poor solvent (χ = 2), the *Q*(ψ) curves become sigmoidal. At low potentials, the brush remains collapsed and stores little charge, yielding a baseline-like slope. With increasing ψ, electrostatics overcome hydrophobic collapse, causing the brush to swell and absorb counterions. This swelling transition produces a pronounced increase in slope—hence a capacitance peak. Once fully swollen, *Q*(ψ) again approaches the good-solvent behavior. In simple terms, the mobile ions in the brush must balance the combined charge of the surface and the charged brush segments; when the brush is collapsed, limited free volume restricts counterion accommodation, leading to complex charge-density distributions and a sharp swelling transition.

For the strongly hydrophobic case (χ = 4), the charge-potential curves show a stepwise growth with pronounced jumps. Between jumps the brushes remain fully collapsed, and the slope is nearly identical to the bare-electrode line. The jumps themselves reflect discrete restructuring events within the collapsed slab and are associated with very sharp, almost divergent capacitance peaks. Hysteresis appears in both salinity conditions (*c*_s_ = 0.4 and 1.6 mol/L), indicating multiple metastable collapsed and partially swollen states. This behavior suggests that swelling proceeds in a layer-by-layer fashion: an outer corona swells first, while the dense inner core yields only at higher potentials.

#### Summary of Charging Regimes

These results illustrate the competition between hydrophobic collapse and electrostatic swelling. At low ψ, hydrophobic interactions dominate and the brush remains compact. At intermediate ψ, Coulomb repulsion drives swelling, producing a strong capacitance enhancement. At high ψ, saturation is reached and the capacitance converges toward the bare-electrode limit. Solvent quality (χ) and ionic strength (*c*_s_) thus provide powerful levers to tune the charging behavior of polymer-brush-modified electrodes.

#### Representative Conformations

Figure 5 presents the microscopic origin of the behaviors observed in Figure 4. The *Q*(ψ) curves in the top row are identical to those in Figure 4 and are reproduced solely to indicate the potentials at which representative conformation snapshots were extracted (white markers).

**Figure 5:**
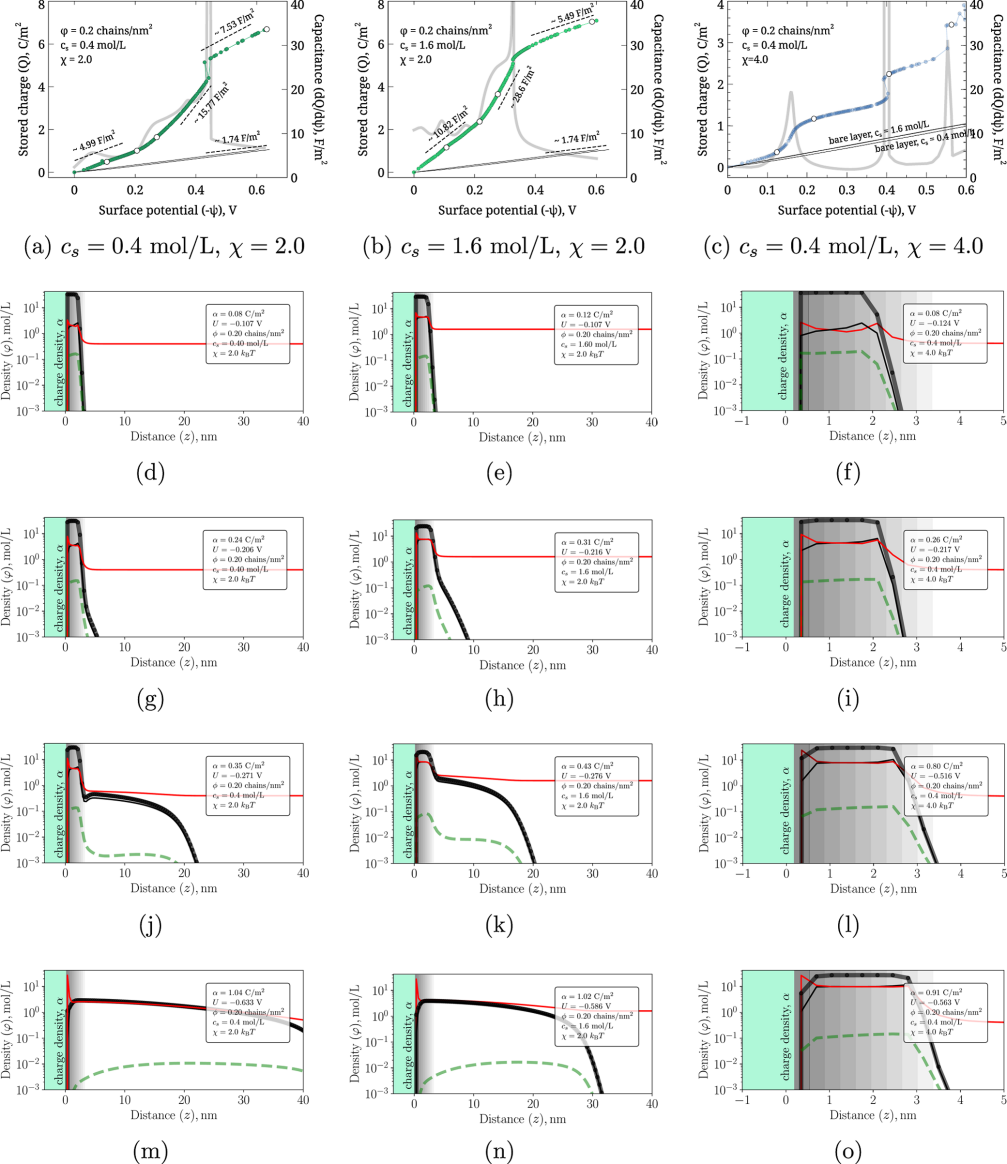
Representative conformations of polymer-brush-modified electrodes. Top row: *Q*(ψ) for the three selected cases (duplicating Figure 4 for convenience). Gray lines show the differential capacitance *C*(ψ). White circles mark the potentials corresponding to the density profiles shown below. Increasing surface potential erodes the collapsed core (gray band), leading to brush swelling and enhanced capacitance.

Across all three cases, the brush initially forms a dense collapsed slab near the electrode. As ψ increases, charged segments accumulate and counterions invade the brush, gradually eroding this slab and increasing the brush thickness. This restructuring coincides with the steepest rise of *Q*(ψ) and yields the large capacitance peaks (15–30 F/m^2^).

For χ = 2.0, the swelling transition is continuous: the brush evolves smoothly from a collapsed core plus swollen corona to a uniformly swollen state. Accordingly, the sigmoidal *Q*(ψ) curve exhibits a single broad capacitance maximum.

For χ = 4.0, the transition is discontinuous. Each jump in *Q*(ψ) corresponds to the collapsed core extending outward by roughly one lattice layer while slightly decreasing in density, allowing counterions to occupy the newly accessible region. Between jumps, the brush remains fully collapsed, giving a bare-electrode-like capacitance. This layer-by-layer erosion explains the piecewise-linear growth and hysteresis seen in Figure 4. It is important to distinguish here between the classical electric double-layer (EDL) capacitance, which remains confined to the narrow near-surface region, and the pseudocapacitance-like behavior arising from the volumetric counterion uptake and redox charging of the entire brush layer. The large capacitance peaks reported here result from the synergy between these two mechanisms, specifically triggered by the conformational restructuring of the brush.

### Physical Picture and Experimental Relevance

#### Volumetric Charge Storage

Our results demonstrate that electroconductive polymer brushes can store substantially more charge than a bare planar electrode. Volumetric counterion uptake, brush swelling, and redox-mediated electron hopping transform the interfacial Helmholtz layer into a nanoscale charge reservoir. At a grafting density of ϕ approximately 0.20 chains/nm^2^, the total stored charge exceeds the surface charge by nearly an order of magnitude, depending on the chain length *N*, consistent with the behavior of pseudocapacitive conducting polymer films.

#### Mechanistic Origin of Performance

The improved electrochemical response emerges from four interacting processes: (i) swelling expands ion-accessible volume; (ii) electron hopping delocalizes charge; (iii) Donnan partitioning drives Na^+^ uptake and Cl^–^ exclusion; and (iv) collapse–swelling transitions produce the observed capacitance maxima. This dual EDL-pseudocapacitive behavior is intrinsic to brush-based architectures. While swelling facilitates counterion access, it also increases the path length for ion transport; however, the simultaneous reduction in brush density and tortuosity likely compensates for this, potentially maintaining high-rate ion mobility compared to dense polymer films.

#### Serial Capacitor Model

The capacitance maximum arises when the collapsed inner core rapidly erodes. In this regime, small increases in ψ trigger large conformational changes and strong counterion uptake. Conceptually, the interface behaves as two capacitors in series: a field-dependent brush capacitor and a Helmholtz capacitor at the electrode. At low ψ, the collapsed brush imposes a large free-energy penalty for ion insertion, which limits the overall capacitance. At intermediate ψ, this penalty decreases as the brush swells, producing a pronounced increase in capacitance. At high ψ, the brush becomes fully swollen and the system approaches the Helmholtz baseline determined by eq 14.

#### Influence of Solvent Quality

The Flory–Huggins parameter χ determines the balance between hydrophobic collapse and electrostatic swelling. In good solvent (χ = 0), brushes remain swollen and provide enhanced capacitance across the full voltage window allowed for aqueous electrolytes. In poorer solvents (χ = 2–4), charging triggers a collapse–swelling transition that generates sigmoidal *Q*(ψ) curves and sharp capacitance peaks (reaching 15–30 F/m^2^), matching experimental reports for PANI and PEDOT coatings. These peaks arise when the dense inner core erodes, enabling rapid structural rearrangements in response to small voltage changes.

#### Redox-Mediated Charge Distribution

Electron exchange with the electrode and intrachain hopping distribute charge throughout the brush, consistent with polaronic transport in conducting polymers.^30^ Although our model assumes fast redox equilibrium, it captures the essential coupling between electronic delocalization and brush swelling.

#### Experimental Comparisons

The coexistence of collapsed and swollen domains resembles the phase heterogeneity observed experimentally in PANI and PEDOT films.^52,53^ Solvophilic modifications—such as cellulose-supported PANI^20^—likewise promote swollen conformations and higher conductivity. Our predicted area capacitances (15–30 F/m^2^) exceed those of even high-surface-area PANI electrodes, which typically achieve only approximately 1–2 F/m^2^ after normalization by geometric surface area, consistent with high-surface-area PANI electrodes reported by Xu et al.^54^

#### Mechanical Stability

The structural transitions predicted here, while significant in volume, are potentially less damaging to electrode durability than the bulk volume changes observed in conventional conductive polymer films. In bulk systems, repeated doping-induced swelling often leads to mechanical fatigue and degradation. In contrast, the brush architecture—with chains tethered at only one end—allows for more flexible conformational changes with lower internal mechanical stress. This structural resilience may offer significant advantages for the long-cycle-life operation of supercapacitors.

#### Future Outlook

By combining fast double-layer charging with redox activity, electroconductive polymer brushes bridge the gap between carbon electrodes and pseudocapacitive polymers. Their high ion capacity and selectivity also suggest potential for electrochemical ion separations.

#### Kinetic and Ohmic Limitations

The SF-SCF framework used here is strictly equilibrium and time-independent: electron transfer, intrachain hopping, and ion partitioning are assumed to be instantaneous, so the applied surface potential coincides with the local redox-equilibrium potential and no overpotential appears. In real electrodes, finite charge-transfer kinetics, electronic resistance of the polymer backbone, and ionic transport resistance through the brush and electrolyte bulk generate activation and ohmic overpotentials. Consequently, the large, sharp capacitance maxima predicted for brushes undergoing collapse–swelling transitions represent thermodynamic equilibrium upper bounds. At finite current or high scan rates, these peaks are expected to broaden, decrease in magnitude, and shift in potential.

#### Limitations of the Mean-Field Approach

As a mean-field theory, SF-SCF neglects explicit ion–ion correlations, finite ion size effects, and dielectric decrement. At the high surface charges and ionic strengths where capacitance enhancement occurs, these effects could be significant. For example, a decrease in the local dielectric constant of water (ε approximately 80) to lower values (ε approximately 5–10) within the collapsed brush core would enhance electrostatic repulsion between segments, likely lowering the potential required for the swelling transition. While these nonidealities might quantitatively shift the predicted capacitance response, the qualitative trends—especially the charging-induced swelling and volumetric storage—remain robust descriptors of the brush physics. Future work should incorporate correlated electrolytes, time-dependent charging, and brush polydispersity to further refine these predictions.

## Conclusions

In this work, we applied the Scheutjens–Fleer self-consistent field method to study electroconductive polymer brushes grafted to planar electrodes in aqueous electrolyte. The simulations reveal that

- Electroconductive polymer brushes amplify charge storage compared to bare electrodes by coupling polymer swelling, ion partitioning, and redox-mediated electron hopping.
- Solvent quality governs a transition from collapsed to swollen states, producing sigmoidal *Q*(ψ) curves and sharp peaks in differential capacitance.
- Grafting density and intrinsic charging propensity (p*K*) tune both the magnitude and voltage range of capacitance enhancement.
- The model reproduces experimental trends, such as high capacitance of polyaniline brushes and conductivity gains from hydrophilic modifications.
- Ion selectivity within the brush highlights potential for electrodialysis and desalination applications, in addition to energy storage.

These results establish hydrophilic electroconductive polymer brushes as a versatile architecture that unites pseudocapacitive redox storage with volumetric ion uptake. By adjusting brush density, solvent compatibility, and redox activity, capacitance values of 200–500 F/g appear achievable within safe aqueous voltage windows. Beyond supercapacitors, the ion-partitioning characteristics of brushes suggest applications in water purification and ion-selective membranes.

Future work should incorporate ion–ion correlations, dynamic transport, and kinetic electron-hopping effects, and validate predictions against experiments with well-defined brush systems. Specifically, the electrochemical quartz crystal microbalance (EQCM) can monitor mass and solvent changes (i.e., changes in the dissipation factor of the oscillation) during the predicted swelling transition,^55,56^ whereas electrochemical atomic force microscopy (EC-AFM) within a liquid environment can directly measure the corresponding change in brush thickness and surface morphology.^57–60^ Furthermore, the structural and charge-distribution changes should produce characteristic signatures in electrochemical impedance spectroscopy (EIS), such as extrema in low-frequency capacitance and shifts in charge-transfer resistance, providing a path for direct experimental verification.^61,62^ Together, these directions will advance polymer-brush-modified electrodes toward practical deployment in sustainable electrochemical devices.

## Data Availability

All simulation data, SF-SCF parameter sets, and analysis routines are provided in a public GitHub repository. The repository includes a Jupyter notebook that reproduces all figures and calculations reported in this manuscript. Repository link: github.com/helvrud/sf-scf-brush-supercapacitor.
